# The Tandem CARDs of NOD2: Intramolecular Interactions and Recognition of RIP2

**DOI:** 10.1371/journal.pone.0034375

**Published:** 2012-03-28

**Authors:** Veronica Fridh, Katrin Rittinger

**Affiliations:** Division of Molecular Structure, MRC-National Institute for Medical Research, London, United Kingdom; Griffith University, Australia

## Abstract

Caspase recruitment domains (CARDs) are homotypic protein interaction modules that link the stimulus-dependent assembly of large signaling platforms such as inflammasomes to the activation of downstream effectors that often include caspases and kinases and thereby play an important role in the regulation of inflammatory and apoptotic signaling pathways. NOD2 belongs to the NOD-like (NLR) family of intracellular pattern recognition receptors (PRR) and induces activation of the NF-κB pathway in response to the recognition of bacterial components. This process requires the specific recognition of the CARD of the protein kinase RIP2 by the tandem CARDs of NOD2. Here we demonstrate that the tandem CARDs of NOD2 are engaged in an intramolecular interaction that is important for the structural stability of this region. Using a combination of ITC and pull-down experiments we identify distinct surface areas that are involved in the intramolecular tandem CARD interaction and the interaction with the downstream effector RIP2. Our findings indicate that while CARDa of NOD2 might be the primary binding partner of RIP2 the two CARDs of NOD2 do not act independently of one another but may cooperate to from a binding surface that is distinct from that of single CARDs.

## Introduction

Pattern recognition receptors (PRRs) are utilized by the innate immune system for the detection of invading pathogens and danger signals. Detection is based on the recognition of specific, evolutionary conserved molecular patterns associated with pathogens or danger signals in the extracellular space and the cytoplasm. Extracellular PRRs include the Toll-like receptors (TLRs) that are also found on lysosomes and endosomes, while intracellular PRRs encompass the NOD-like receptor (NLR) and RIG-I like receptor (RLR) families [1]–[3]. Members of the NLR family contain a tri-partite domain structure with a C-terminally located ligand binding domain (LBD) that consists of a varying number of leucine rich repeats (LRRs). These are flanked by a centrally located NACHT domain (also referred to as NOD, nucleotide-binding and oligomerization domain), which oligomerizes in a ligand and nucleotide dependent fashion to expose an N-terminally located effector binding domain (EBD) that mediates the interaction with downstream effectors to induce activation of specific signaling processes [4]–[6].

The interaction of NLRs and RLRs with downstream effectors is mediated by members of the death fold superfamily, a family of protein interaction modules that comprises 4 subfamilies: death domains (DD), death effector domains (DED), caspase recruitment domains (CARDs) and pyrin domains (PYD) [7], [8]. Members of this protein family form homotypic interactions and play important roles in the regulation of inflammatory and apoptotic signaling pathways, often by promoting the assembly of large multi-protein complexes [9]–[11]. In general, members of this superfamily share low sequence homology but adopt a similar compact, globular fold consisting of a six helix bundle called the death domain fold [7].

Complexes formed between members of the death domain subfamily are structurally the best characterized and show how a given DD is capable of simultaneously engaging up to six binding partners using three different types of homotypic interactions referred to as types I, II and III [7], [8], [12]. Type I interactions are represented by the complex between the CARDs of Apaf-1 and procaspase-9, whose crystal structure revealed an interface involving charge-charge interactions between helices α2 and α3 of Apaf-1 and helices α1 and α4 of caspase-9. In addition, the interacting surface areas of each protein have a complimentary shape [13]. A similar mode of interaction has been suggested to occur between other CARD-CARD complexes [14]. Type II interactions have first been found in the DD-DD complex formed between Pelle and Tube [15], and involve helix α4 and the loop connecting helices α4 and α5 of one domain and the loop connecting α5 and α6 plus helix α6. Type III interactions have not been observed in dimeric complexes but exist in the structures of the PIDDosome, the MyDDosome and the Fas/Fadd DISC [16]–[18]. The PIDDosome core complex is formed by five PIDD DDs and seven RAIDD DDs, in which all DDs exist in a quasi-equivalent environment and all 3 types of interfaces occur. Similarly the MyDDosome, formed by MyD88, IRAK-4 and IRAK-2 shows all three types of interactions to occur suggesting that they constitute a common mode of interaction within the DD superfamily.

**Figure 1:**
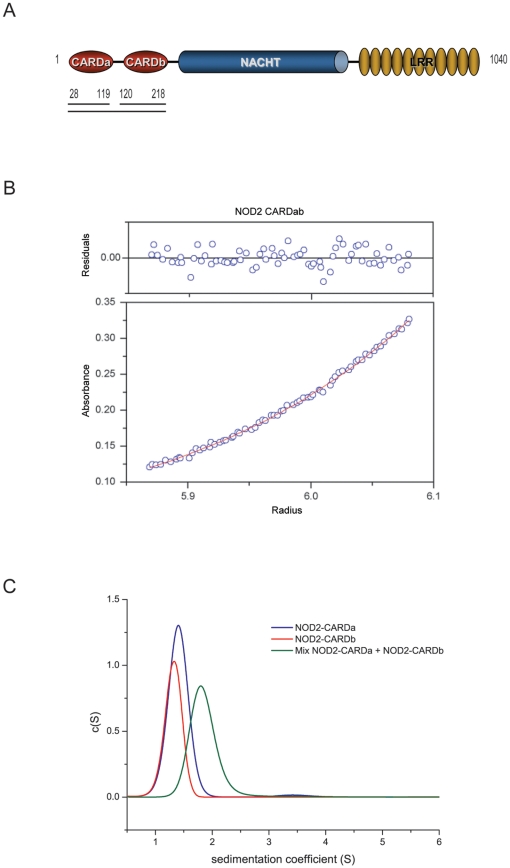
Oligomeric state of the CARDs of NOD2. (A) Schematic representation of the domain structure of human NOD2 and the domain boundaries of the constructs (tandem CARD, CARDa and CARDb) used in this study. (B) AUC Sedimenation Equilibrium resulted in MW of 22 kDa±0.5 kDa for CARDab (calculated MW = 22043 Da). The sample was run at three different concentrations and three different speeds (18, 22, 26 kprm). 11 scans were collected over 4 days. (C) AUC Sedimentation Velocity resulted in MW of 10–11 kDa for CARDa (blue line, calculated MW = 11155 kDa) and CARDb (red line, calculated MW = 11403 kDa), respectively. Samples were run at OD_600_∼0.5. SedFit was used for data analysis. A sample containing equal molar amounts of CARDa and CARDb displayed a MW of ∼18 kDa.

At present DDs constitute the only subfamily that has been shown to be able to promote the formation of large multi-protein assemblies through homo-oligomerization. In contrast, the CARD and pyrin domain-containing NLR proteins are believed to form higher order complexes via oligomerization of their NACHT domains, while the CARDs or PYDs are assumed to interact in a 1∶1 fashion with their downstream effectors [7]. Unlike other NLRs, NOD2 a protein that regulates nuclear factor κB (NF-κB) activation and mutations in which have been linked to a predisposition to Crohn's disease [19], [20] contains 2 CARDs in tandem (Figure 1A). Ligand sensing by the LRRs of NOD2 and subsequent NACHT domain-mediated self-oligomerization is thought to induce the recruitment and polyubiquitination of the downstream effector kinase RIP2 that in turn activates the NF-κB signaling pathway and subsequent transcription of proinflammatory genes [21], [22]. Complex formation between NOD2 and RIP2 relies on the specific recognition of their respective CARDs, an interaction which is not understood on a molecular level.

Here we provide the first biophysical characterization of the tandem CARDs of NOD2. Our study uncovered that the two CARDs interact with one another in an intramolecular fashion. We present a biophysical analysis of the isolated and tandem domains, which is aimed at understanding the molecular basis of this intramolecular interaction and provide data from a mutational analysis that suggest that different sites are used for the intra- and intermolecular CARD-CARD interactions of NOD2.

## Results and Discussion

### Oligomeric state

The tandem CARDs of NOD2 (Figure 1A) were overexpressed as a construct comprising amino acids 28–218 that was stable and monodisperse by dynamic light scattering (DLS). In contrast, expression of isolated CARDa could not be achieved in *E.coli*, despite re-synthesis of the gene optimized for codon-usage in bacteria and testing of different domain boundaries and bacterial strains for expression. Therefore, to overcome expression problems the individual CARDs were purified from a modified version of the GST-tagged tandem CARD construct. A thrombin-cleavage site consisting of 5 amino acid residues (VPRGS) was inserted between CARDa and CARDb at amino acid Leu119. The resulting GST-fusion protein was cleaved twice during affinity chromatography, first with thrombin to release CARDb and then with Precission protease to generate CARDa. Surprisingly, after cleavage of the linker connecting CARDa and CARDb, a large proportion of the two domains stayed associated, suggesting that they may be engaged in an intramolecular interaction.

To assess potential self-association of the tandem as well as individual CARDs of NOD2 we used equilibrium and sedimentation analytical ultracentrifugation (AUC), respectively. The equilibrium experiment indicated that the tandem CARD construct of NOD2 is monomeric with no tendency to associate into higher order oligomers at the concentrations tested (Figure 1B). Similarly, sedimentation velocity AUC of the individual CARDs showed that either domain exists as a monomer in solution (Figure 1C). However, analysis of a sample containing equimolar amounts of CARDa and CARDb led to the formation of a new species with a higher sedimentation coefficient, indicating that the two CARDs form a 1∶1 complex (Figure 1C). Although these experiments indicate that the CARDs of NOD2 do not have a high propensity to self-associate, neither as individual domains nor as the tandem construct, we noticed that at high protein concentrations (0.5–1.0 mM) there is a tendency of the tandem as well as isolated CARD constructs to self-associate as observed in ^15^N HSQC NMR spectra (unpublished data), in line with the reported homodimerization of a tandem CARDab construct as seen by yeast two-hybrid analysis [23]. Taken together, these data indicate that the individual CARDa and CARDb domains interact with each other in solution, even in the absence of a linker, and that the tandem CARDs have a tendency to form homo-oligomers at high protein concentrations.

### Fold and stability

**Figure 2:**
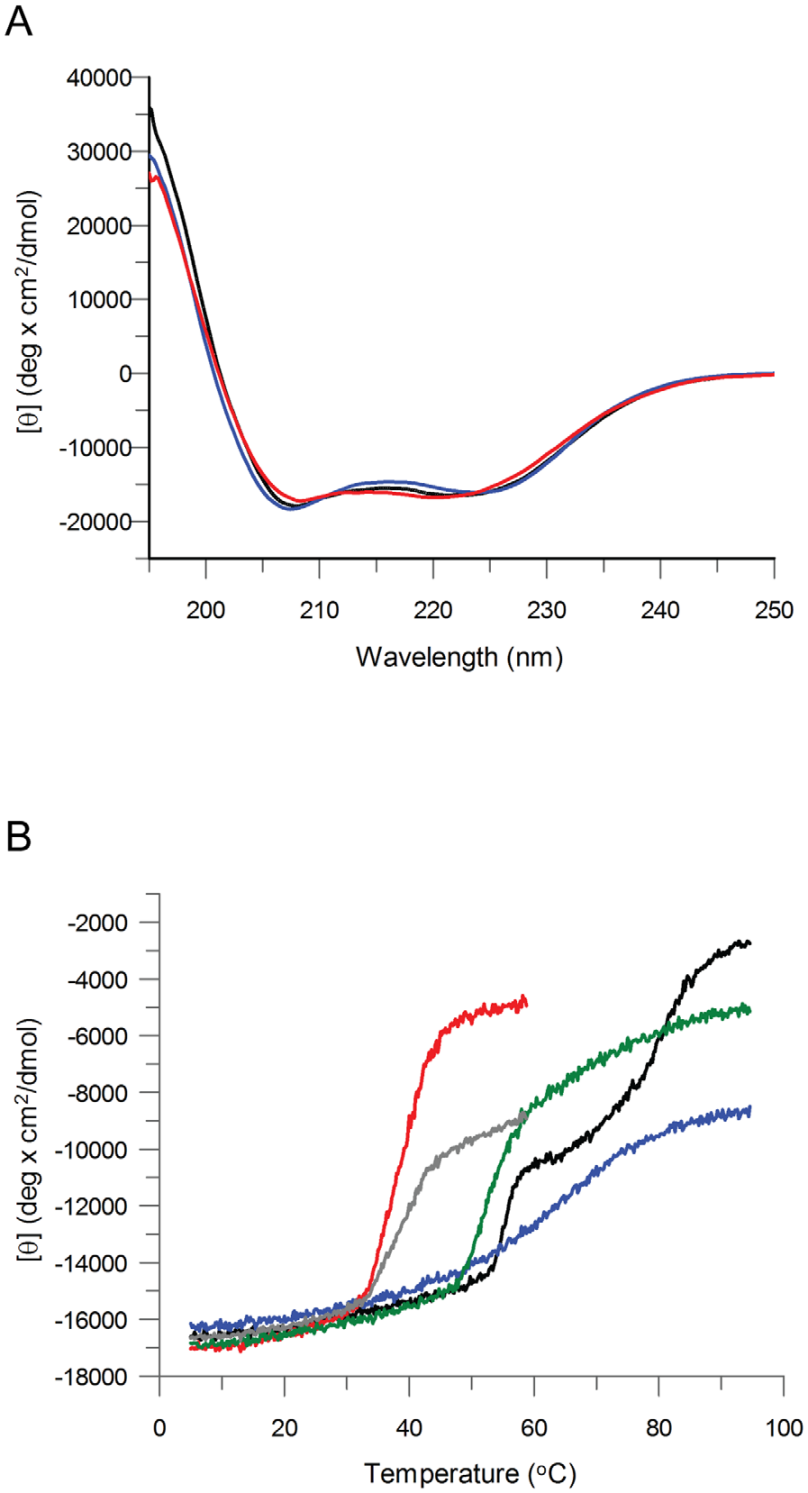
Fold and stability of the CARDs of NOD2. (A) Far-UV CD at 250–195 nm showed highly α-helical proteins as reflected in the double minima at 208 and 222 nm and the strong positive band at 195 nm. Sample concentration was 0.15 mg/ml. The black curve shows the mean residue ellipticity of NOD2-CARDab, the blue curve of NOD2-CARDa and the red curve of NOD2-CARDb. (B) CD thermal unfolding from 5 to 95°C at 222 nm. Sample concentration was 0.15 mg/ml. A 2 mm cuvette was used. The black curve represents the mean residue ellipticity of NOD2-CARDab, the blue curve of CARDa and the red curve of CARDb. The mixture contained 0.075 mg/ml of NOD2-CARDa and NOD2-CARDb, respectively, and is shown in green. The grey curve represents the computed mean value of NOD2-CARDa and NOD2-CARDb.

To test if CARDa and b act as independent domains or might stabilize each other we analyzed their fold and stability by circular dichroism (CD), in tandem and as individual domains. The far-UV spectra of the individual and tandem domains (Figure 2A) confirmed the high α-helical content that was expected based on available crystal and NMR structures of other CARDs. Stability analysis was carried out by thermal unfolding of the proteins at 222 nm between 5 and 95°C (Figure 2B). Isolated CARDa unfolded in a reversible manner with a T_m_ = 66.2°C (blue curve) and CARDb in a non-reversible manner at T_m_ = 38.0°C (red curve). A comparison of the melting temperatures of the individual domains to those of other CARDs including NOD1 CARD (T_m = _78.0°C), Apaf-1 CARD (T_m = _59.7°C) and procaspase-9 CARD (T_m = _53.4°C) (data not shown) highlights that CARDb unfolds at a particular low temperature. In contrast, the tandem CARDs of NOD2 unfolded in two non-reversible transitions at T_m1_ = 55.6°C and T_m2_ = 80.8°C (black curve). Similarly, a reconstituted complex prepared by mixing equimolar amounts of CARDa with CARDb underwent two transitions (Figure 2B, green curve). The first transition occurred at a similar temperature to that of the tandem construct, around 55°C while the second transition was observed at a lower temperature than the tandem construct, but above 60°C. Interestingly, the first transition in the reconstituted complex as well as in the tandem CARD protein took place at a higher temperature compared to the computed mean value of the individual CARDa and CARDb curves (grey curve), indicating that the more unstable domain, CARDb is stabilised by CARDa in both cases. Furthermore, the second transition in the tandem CARD construct was observed at a much higher temperature compared to that of the reconstituted complex, suggesting that the covalent link might provide an additional level of stabilization. Nevertheless, the similar two-step thermal unfolding curves obtained for the reconstituted complex and the tandem construct strongly suggest that CARDa and CARDb interact in the context of the covalently linked wild-type protein and not only after removal of the linker.

### Thermodynamics of the intramolecular CARD-CARD interaction

**Figure 3:**
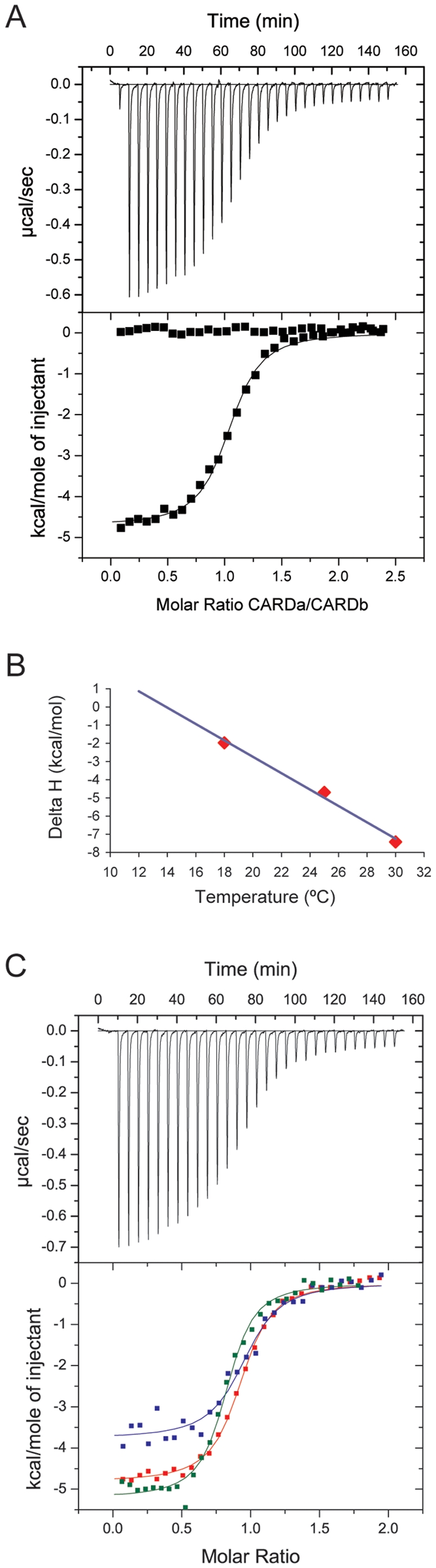
Thermodynamics of the NOD2 CARDa-CARDb interaction. (A) ITC measurement of complex formation between CARDa in the syringe (475 µM) and CARDb in the cell (45 µM). T = 25°C. The binding isotherm was fitted to a one-site binding model with a K_d_ of 1.1 µM. A control experiment of CARDa into buffer is shown. (B) Determination of the heat capacity, ΔC_p_. Enthalpies, ΔH, from CARDa-CARDb titrations at different temperatures were plotted against the temperatures. Linear regression analysis gave ΔC_p_ = dΔH/dT = −450 cal/(mole °C). (C) Effect of CARDa point mutations as monitored by ITC at 25°C. Titration of CARDa E69K (345 µM) into CARDb (40 µM) is shown in blue, CARDa E72K (205 µM) into CARDb (26 µM) in green and CARDa R86A (504 µM) into CARDb (62 µM) in red. The titrations were performed in the same buffer as in (A).

To quantify the interaction between CARDa and CARDb we carried out isothermal titration calorimetry (ITC) experiments. Titration of CARDa into CARDb at 18, 25 and 30°C resulted in binding curves that could be fitted to a single site binding model with a dissociation constant, K_d_ of 0.9–1.4 µM and a stoichiometry of 1∶1 (Figure 3A, Table 1). The heat capacity of complex formation ΔCp was determined to be −450 cal/(mole °C) (Figure 3B). The linear line indicates that the CARDa-CARDb interaction is unlikely to be affected by temperature dependent processes such as thermal unfolding of the proteins, temperature-induced changes in native protein conformation or changes in aggregation state [24].

**Table 1:** Thermodynamic parameters for the CARDa-CARDb interaction.

Injectant	T	N	K_d_	ΔG	ΔH	TΔS
	(°C)		(µM)	(kcal mol^−1^)	(kcal mol^−1^)	(kcal mol^−1^)
CARDa wt	18	0.84	1.08	−7.9	−2.0	5.9
CARDa wt	25	1.03	0.88	−8.3	−4.7	3.6
CARDa wt	30	0.91	1.41	−8.1	−7.4	0.7
CARDa E69K	25	0.94	0.72	−8.4	−3.8	4.6
CARDa E72K	25	0.81	0.33	−8.8	−5.2	3.6
CARDa R86A	25	0.91	0.81	−8.3	−4.8	3.5

Previous studies on CARD-CARD complexes reported complex formation to be driven, at least in part, by charge-charge interactions [13]–[15]. To test whether the interaction between CARDa and CARDb of NOD2 has a similar electrostatic component, ITC titrations were performed at increasing NaCl concentrations (Table 2). The interaction was weakened but not disrupted at higher NaCl concentrations, from K_d_ = 1.1 µM at 50 mM NaCl to K_d_ = 7.1 µM at 1000 mM NaCl (at 30°C). The change in enthalpy, ΔH, decreased at higher salt concentrations (from −9.0 kcal/mol to −2.6 kcal/mol), which is likely to reflect the breaking of salt bridges. In contrast, the change in entropy, TΔS, increased at higher salt concentrations (from −0.7 cal/(mol*K) to 4.6 cal/(mol*K)) reflecting the increase in disorder which is likely to originate from a gain in translational and rotational freedom of protein side chains as interactions are disrupted. These data suggest that the intramolecular CARDa-CARDb interaction has indeed an electrostatic contribution similar to that observed for intermolecular interactions, but that van der Waals interactions are also part of the interface.

**Table 2:** Salt dependence of the CARDa-CARDb interaction.

Injectant	NaCl	N	K_d_	ΔG	ΔH	TΔS
	(mM)		(µM)	(kcal mol^−1^)	(kcal mol^−1^)	(kcal mol^−1^)
CARDa wt	50	0.61	1.09	−8.3	−9.0	−0.7
CARDa wt	100	0.91	1.41	−8.1	−7.4	0.7
CARDa wt	250	0.99	2.99	−7.7	−5.5	2.2
CARDa wt	500	0.85	6.25	−7.2	−4.8	2.4
CARDa wt	1000	0.78	7.14	−7.1	−2.6	4.5

ITC measurements were performed at 30°C in 50 mM Tris-HCl, 2 mM DTT, pH 7.5 with the ionic strength ranging from 50–1000 mM NaCl. Sample concentrations were 430–594 µM in the syringe (CARDa) and 38–52 µM in the cell (CARDb). The low n-value (N = 0.6) obtained at 50 mM NaCl may reflect that CARDb is partially unfolded at this NaCl concentration.

### Intra- versus intermolecular CARD-CARD interactions

Our ITC experiments demonstrate that the CARDs of NOD2 interact with a 1∶1 stoichiometry and an affinity of around 1 µM, comparable to some of the published dissociation constants for homotypic interactions within the DD superfamily: the RIP2-caspase1 CARD-CARD complex; K_d_ of 1.4 µM [25], the Pelle-Tube DD-DD complex; K_d_ of around 0.5 µM [26], [27] and the ASC-Pop1 PYD-PYD complex; K_d_ of 4 µM [28]. In addition, our own ITC experiments gave a K_d_ of ∼1–4 µM for the CARD-CARD interaction between Apaf-1 and procaspase-9 at different temperatures (data not shown).

The fact that CARDa and CARDb are covalently linked in NOD2 but are able to interact with each other with an affinity similar to that determined for intermolecular CARD-CARD complexes is intriguing and raises the question if the binding sites for interaction with downstream effectors such as RIP2 and each other are distinct or possibly overlapping and hence must be subject to some kind of regulatory event. To address this question, we carried out a mutational analysis of the tandem CARDs in order to determine the surface areas involved in either interaction. Mutations were designed based on the structure of the Apaf-1/procaspase-9 CARD-CARD complex [13] and a mutational study of the NOD1 and RIP2 interaction [14] and acidic as well as basic residues in each CARD were targeted. Seven mutations were introduced into the modified tandem CARD construct (D58A, E69K, D70A, E72K and R86A in CARDa and D154A and R182A in CARDb) but only three expressed in sufficient quantities to allow analysis by ITC (E69K, E72K and R86A). Interestingly, neither of these mutations in CARDa significantly affected the strength of the interaction indicating that the targeted residues are not part of the intramolecular CARD-CARD interface (Figure 3C, Table 1).

**Figure 4:**
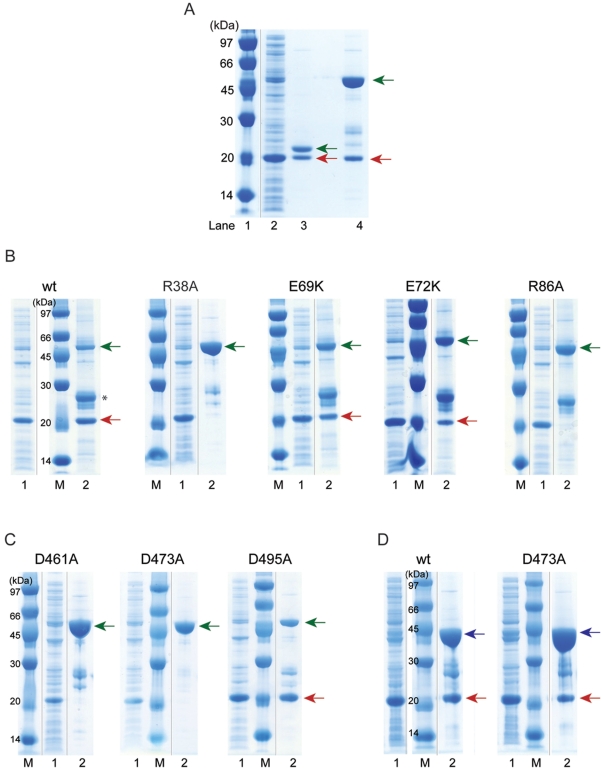
Complex formation between NOD2 CARDab and RIP2-CARD. (A) NOD2 CARDab with a N-terminal GST-tag and RIP2-CARD equipped with a N-terminal GB1-tag and a C-terminal His_6_-tag were co-expressed and pulled-down with glutathione sepharose beads. From left: Lane 1) Protein Marker, GE Healthcare. Lane 2) Soluble lysate. Lane 3) Bead eluate after 3C-protease cleavage. NOD2-CARDab 22.0 kDa and GB1-RIP2 CARD-His 18.6 kDa are indicated by green and red arrows, respectively. Lane 4) Supernatant bound to beads. GST-NOD2 CARDab 48.5 kDa (green) and GB1-RIP2 CARD-His 18.6 kDa (red) are indicated by arrows. (B) Effect of NOD2 CARDab single point mutations on RIP2 CARD binding. A representative cross section of the mutants tested are shown. GST-NOD2 CARDab 48.5 kDa and GB1-RIP2 CARD-His 18.6 kDa are indicated by green and red arrows, respectively. * = residual expression of GST. (C) Effect of RIP2 CARD single point mutations on NOD2 CARDab binding. A cross section of the mutants tested are shown. GST-NOD2 CARDab 48.5 kDa and GB1-RIP2 CARD-His 18.6 kDa are indicated by green and red arrows, respectively. (D) Effect of RIP2 CARD single point mutations on NOD1 CARD binding. A cross section of the mutants tested are shown. GST-NOD1 CARD 40.9 kDa (aa17–138) and GB1-RIP2 CARD-His 18.6 kDa are indicated by blue and red arrows, respectively.

To investigate if these amino acids might instead be important for the interaction with the downstream effector RIP2, we attempted to produce a construct of the RIP2 CARD that would be suitable for ITC studies. Unfortunately though, all tested RIP2 CARD constructs proved to be insoluble. Refolding procedures allowed us to prepare a protein that contained the expected helical content as shown by CD spectroscopy but surprisingly was not able to bind to NOD2 CARDab suggesting that the refolded protein may not have adopted the correct tertiary structure, an interpretation that is supported by 1D(^1^H)-NMR spectroscopy (data not shown). Therefore, we decided to study the intermolecular NOD2-RIP2 CARD-CARD interaction by co-expression in *E.coli* followed by pull-down experiments. Co-expression of the GST-tagged tandem CARDs of NOD2 together with the CARD of RIP2 (aa435–528) fused to an N-terminal solubility enhancement tag, the GB1-tag (protein G B1 domain) and a C-terminal His-tag followed by pull-down with glutathione sepharose beads clearly showed a NOD2-RIP2 interaction (Figure 4A). Control experiments confirmed that there is no unspecific interaction between RIP2-GB1 and GST (Figure S1A) or between the GB1-tag and GST-NOD2-CARDab (Figure S1B). Furthermore, the co-expression system showed a direct interaction between NOD1-CARD (aa17–138) and RIP2-CARD (see below) but, as a control, not between caspase-9 CARD (aa1–112) and RIP2-CARD demonstrating that RIP2 CARD does not interact unspecifically with other CARDs in our assay.

### Mutagenesis of NOD2-CARDab and RIP2-CARD

**Figure 5:**
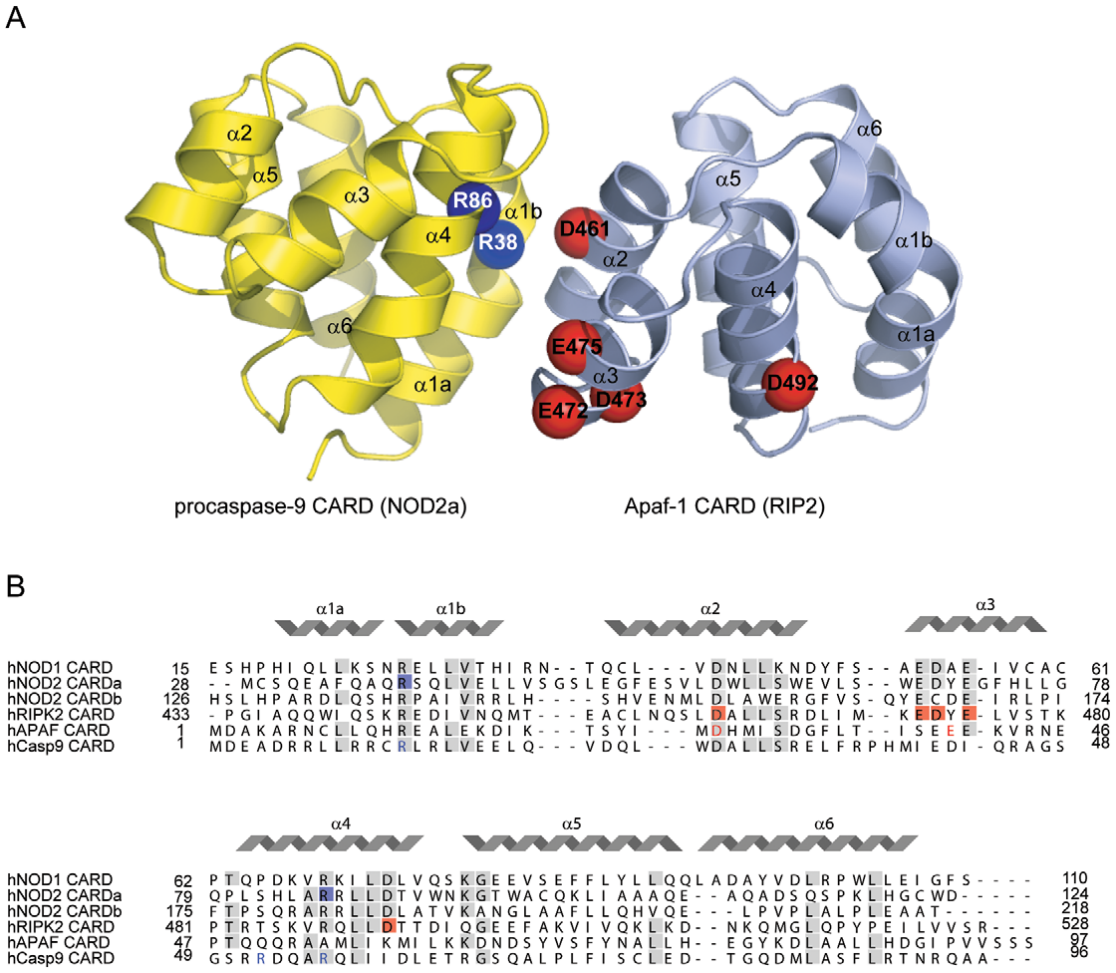
Residues involved in the NOD2-RIP2 interaction. (A) Structure of the CARD-CARD complex between Apaf-1 (light blue) and procaspase-9 (yellow), pdb ID 3YGS. The relative location of residues that were identified to disrupt the NOD2-RIP2 interaction has been mapped onto the CARD-CARD structure, based on the alignment shown in (B). These include R38 and R86 (shown in dark blue) located in CARDa of NOD2 that are shown mapped onto the CARD of procaspase-9 and D461, E472, D473, E475 and D492 in RIP2 (shown in red), mapped onto the CARD of Apaf-1. (B) The featured residues are highly conserved in CARDs. CARDa R38 and R86 correspond to two (R13 and R56) of the three basic residues in procaspase-9 (shown in blue) that are crucial for the interaction with Apaf-1. CARDa has no equivalent to the third residue, R52. Conversely, RIP2 CARD D461 corresponds to Apaf-1 D27 and RIP2 E472, D473 and E475 are located in the region of Apaf-1 E40. Apaf-1 D27 and E40 (shown in red) are both crucial for the interaction with caspase-9.

To investigate the NOD2-RIP2 interaction in more detail and identify the binding regions involved, a number of residues were mutated based on sequence conservation within the CARD family (Figure 5B) and previously reported mutagenesis studies [23], [29]. Single-point mutations introduced into the expression plasmid for wild-type NOD2-CARDab included Q31H, R38A, E69K, E72K, D58A, D70A, R86A, R87A, A106V (in CARDa) and L145P, D154A, Q164K, E166K and R182A (in CARDb). GST pull-down analysis showed that NOD2 CARDab Q31H, E69K, E72K, R87A, A106V, D154A and Q164K could still interact with RIP2 CARD, similar to the wild-type protein. Mutations D58A, D70A, L145P E166K and R182A could not be evaluated due to very low expression levels. In contrast, two mutations, R38A and R86A located in CARDa, abolished the interaction with RIP2 CARD (Figure 4B, Table 3). Assuming a scenario in which NOD2 uses predominantly conserved, basic residues (R38 and R86) to interact with conserved, acidic residues of RIP2, in analogy with other CARD-CARD interactions, six additional point mutations were made in RIP2 CARD. These included D461A, E472A, D473A, E475A, D492A and D495A. Pull-down analysis with the tandem CARDs of NOD2 showed that one mutant, D495A could bind NOD2 as well as wild type while the other five mutants, D461A, E472A, D473A, E475A and D492A disrupted the interaction with NOD2 CARDab (Figure 4C, Table 4). In addition to evaluating the effect of the RIP2 CARD mutants on the interaction with NOD2, all RIP2 mutants were also tested for their ability to bind the CARD of NOD1. These experiments gave the same results as above for five of the mutants: D495A had no effect on binding to NOD1 CARD while D461A, E472A, E475A and D492A disrupted the interaction. However, the RIP2 mutant D473A that disrupted the interaction to NOD2 was able to bind NOD1 CARD (Figure 4D, Table 4). This observation suggests that there are distinct differences in the interaction of RIP2 with NOD1 or NOD2 and that their respective CARDs are not interchangeable.

**Table 3:** The interaction of NOD2 CARDab mutants with the CARD of RIP2.

NOD2 CARDab	RIPK2 CARD
wt	+
Q31H (CARDa)	+
R38A (CARDa)	−
D58A (CARDa)	N/A
E69K (CARDa)	+
D70A (CARDa)	N/A
E72K (CARDa)	+
R86A (CARDa)	−
R87A (CARDa)	+
A106V (CARDa)	+
L145P (CARDb)	N/A
D154A (CARDb)	+
Q164K (CARDb	+
E166K (CARDb)	N/A
R182A (CARDb)	N/A

Presence (+)/absence (−) of RIP2 CARD on the beads following coexpression and GST-pull downs as analyzed by SDS-PAGE. N/D = not determined (no or too low expression for evaluation).

**Table 4:** The interaction of RIP2 CARD mutants with NOD2 CARDab and NOD1 CARD.

RIPK2 CARD	NOD2 CARDab	NOD1 CARD
wt	+	+
D461A	−	−
E472A	−	−
D473A	−	+
E475A	−	−
D492A	−	−
D495A	+	+

Presence (+)/absence (−) of RIP2 CARD on the beads following coexpression and GST-pull downs as analyzed by SDS-PAGE.

Based on our results we suggest a model, in which RIP2 uses an acidic surface for the interaction with both, NOD1 and NOD2. In contrast, a study by Wagner *et al* [23] predicted NOD2 to use an acidic surface to interact with RIP2. In their yeast two-hybrid analysis, two single point mutations in CARDa, E69K and D70A, as well as a triple mutant (E69K, D70A and E71K) in the tandem domain of NOD2 failed to bind RIP2 CARD. In the present study the effect of D70A could not be evaluated due to lack of expression. E69K on the other hand, showed wild type expression levels and wild type ability to interact with RIP2 CARD in the GST-pull down assay implying that E69 does not contribute to the interaction. In another study, Manon *et al* [14] concluded that RIP2 uses a basic surface to bind NOD1. This group used the NMR-structure of NOD1 and a homology model of RIP2 as the basis for mutagenesis and based on co-immunoprecipitation and *in vivo* NF-κB activation assays identified an acidic patch on NOD1 and basic patch on RIP2 as critical for the interaction. It is not clear at present if the apparent differences between those studies and ours are due to the different experimental set-ups used or reflect true differences in the interaction of RIP2 with NOD1 or NOD2 and it will require the structures of the respective RIP2/NOD1 and RIP2/NOD2 complexes to settle this question.

All residues identified in the present study to disrupt the interaction with RIP2 are located in CARDa, suggesting that NOD2 primarily uses this domain for the interaction with RIP2 (Figure 5). Such a model agrees with the findings of Rosenstiel *et al* [30] who showed, based on co-immunopreciptiation data, that a short isoform of NOD2, NOD2-S which covers CARDa and only the first 54 residues of CARDb, is still able to interact with RIP2 CARD. On the other hand a study by Ogura *et al* [31] suggested that overexpression of both CARDs is required to induce NF-κB signaling in the absence of MDP, whereas neither CARDa nor CARDb alone had this effect. Similarly, Wagner *et al* [23] found that both CARDs of NOD2 were required for recruitment of RIP2 CARD in a yeast two-hybrid analysis. However, neither of these studies investigated the molecular basis for the observation that both CARDs seem required for the interaction with RIP2 and asked if the two domains constituted independent binding modules or may require each other for their stability.

We now show that in addition to recognizing RIP2 the tandem CARDs interact in an intramolecular fashion with an affinity that is similar to that identified for other CARD-CARD complexes. Importantly, we have identified a mutation in NOD2 CARDa, R86A that fully disrupts the interaction with RIP2 but has no effect on binding to CARDb. These results strongly indicate that the surfaces used by NOD2 for intra- and intermolecular interactions differ and hence are not mutually exclusive. Furthermore, our thermal unfolding studies showed that the interaction between the two CARDs significantly increases their stability, suggesting that the two domains do not act independently of one another. Based on these observations it is tempting to speculate that NOD2 may use an extended surface created by the interaction between the two CARDs to interact with RIP2 in a manner that is different from typical CARD-mediated interactions, which could explain the discrepancy between our data and those reported for the NOD1-RIP2 interaction.

Mutual stabilization of tandem protein interaction modules, in which only one module may be able to interact with ligands, has been observed in other proteins. For example two of the six PDZ domains of glutamate receptor-interacting protein (GRIP) pack against each other to form a stable, supramodular structure that supports binding of one of the two domains to its target [32]. Similarly, adjacent WW domains in Suppressor of deltex (SU(dx)) interact with one another and this arrangement is stabilized upon ligand binding to one of the tandem domains, while the other is lacking a functional ligand binding site [33]. Furthermore, adjacent protein interaction modules may interact to form a higher order structure that has ligand binding properties that are distinct from those of the individual domains as observed for the tandem SH3 domains of the NADPH oxidase subunit p47^phox^ which form a superSH3 domain that contains only a single ligand binding site [34].

### Conclusions

We provide the first biophysical characterization of the tandem CARDs that constitute the effector binding domain of NOD2. This study led to the discovery of an intramolecular interaction between CARDa and CARDb with a stoichiometry and affinity similar to previously studied intermolecular CARD-CARD interactions but occurs using different sites for intra- and intermolecular interactions. Based on mutational analysis we propose a model in which R38 and R86 located in CARDa of NOD2 interact with a set of acidic residues on RIP2 CARD suggesting that NOD2 might use CARDa as the main binding site for the interaction with RIP2. Whether CARDb participates directly in binding of RIP2 or indirectly, e.g. by exerting an activating or stabilizing effect on CARDa, needs further assessment.

Finally, we noticed that the complex between NOD2 CARDab and RIP2 CARD dissociates after removal of the GST tag and elution from beads indicating that oligomerization of NOD2 might be a prerequisite for the formation of a stable complex with RIP2 and could be mimicked by the GST tag in our experiments. Oligomerization of NLRs is thought to be mediated by their respective NACHT domains and such an event could bring the CARDs into close vicinity and promote an interaction which is otherwise very weak as shown by our AUC and NMR experiments. A high resolution structure of the NOD2-RIP2 complex is now required to fully understand the molecular details of this interaction.

## Materials and Methods

The cDNA of human NOD2 was a kind gift from T. Segal and that of RIP2 from D. Abbott. All other cDNAs were purchased from Source BioScience. Mutagenesis was carried out using the QuikChange XL site-directed mutagenesis kit (Stratagene). DNA sequences of all plasmids were verified by DNA sequencing.

### Expression and purification of the tandem NOD2-CARDab construct

The DNA coding for residues 28–218 of NOD2 was cloned into pGEX-6P1 vector (GE Healthcare). NOD2-CARDab, was expressed in *Escherichia coli* Rosetta2 (DE3) cells (Novagen) and contained a cleavable N-terminal GST-tag. Cells were grown in LB-media at 37°C and protein expression was induced with 0.3 mM IPTG at 18°C o/n. The recombinant protein was purified using Glutahione sepharose 4B (GE Healthcare) at pH 7.5. The GST-tag was removed by Precission protease cleavage o/n. The eluted NOD CARDab protein was further purified to homogeneity on a Superdex75 column (GE Healthcare) and concentrated in VivaSpin concentrators.

### Expression and purification of the individual CARDs

The tandem NOD2-CARDab expression plasmid was modified to allow the expression and purification of the individual CARDs as follows: site-directed mutagenesis was used to insert five residues (VPRGS) between amino acid 119 (L) and 120 (H) thereby creating a thrombin cleavage site between the two individual domains. Expression and purification were performed as described above but with the following modifications: during affinity chromatography the GST-fusion protein was cleaved twice on-column, first with human α-thrombin protease (Cambridge Biosciences) and then with Precission protease to generate NOD2-CARDa and NOD2-CARDb, respectively. An anion exchange step at pH 7.5 (Sepharose Q FF, GE Healthcare) was required to separate the two CARD domains completely prior to size exclusion chromatography. The final NOD2-CARDa and NOD2-CARDb proteins were concentrated to approx. 700 µM and 400 µM, respectively.

Protein identity of NOD2-CARDab, NOD2-CARDa and NOD2-CARDb was verified by electrospray mass spectrometry. Protein concentrations were determined by UV-spectrometry using calculated extinction coefficients.

### Analytical Ultracentrifugation (AUC)

Equilibrium AUC and sedimentation velocity studies were carried out using a Beckman Optima XL-A analytical ultracentrifuge. The NOD2 CARDab tandem protein was subjected to equilibrium AUC using a Beckman An-60 Ti rotor in 50 mM Tris, 150 mM NaCl, 1 mM TCEP, pH 7.5 at three optical densities (0.2, 0.4 and 0.6). Equilibrium was reached at three different speeds (18, 22, 26 kprm) over 4 days. ORIGIN-based XL-A analysis software was used to determine the molecular mass. The CARDa and CARDb protein domains of NOD2 were subjected to sedimentation velocity experiments as individual samples and as a 1∶1 mixture. Experiments were carried out at optical densities of 0.5 at 280 nm. The software SedFit was used for data analysis to determine the sedimentation coefficient *s*, the frictional coefficient *f* and estimation of mass.

### Circular Dichroism (CD) Spectroscopy

CD spectra were recorded with a JASCO-J715 spectropolarimeter. Samples were prepared in 25 mM Tris-buffer pH 8.0. The protein concentration for far-UV and thermal unfolding was 0.15 mg/mL in a 1 mm cuvette. A reconstituted CARDa-CARDb complex was prepared by mixing equimolar amounts of NOD2-CARDa and NOD2-CARDb. Far-UV experiments were run at 20°C. Thermal unfolding/folding was performed at a single wavelength (222 nm) between 5–95°C. All spectra were corrected for buffer signals.

### Isothermal Titration Calorimetry (ITC)

Complex formation between NOD2-CARDa and NOD2-CARDb was measured by ITC using a MicroCal VP-ITC microcalorimeter (GE Healthcare). Samples were dialysed into ITC buffer (50 mM Tris-HCl, 100 mM NaCl, 2 mM DTT, pH 7.5). Typically, the sample cell contained NOD2-CARDb at 40–50 µM and the syringe NOD2-CARDa at 400–500 µM. Titrations with 29 injections of 10 µl were carried out at different temperatures (12, 15, 25, 30°C) and different NaCl concentrations (50–1000 mM at 30°C). Heats of dilution were subtracted from the raw titration data before analysis. Data were fitted by least-square procedures assuming a one-site binding model using Microcal Origin version 7.0.

### Co-expression and GST-pull down experiments

Direct CARD-CARD interactions between NOD2 and RIP2 were studied by co-overexpression in *E.coli* followed by GST-pull down experiments. Plasmids of NOD2 tandem CARD (aa28–218) in pGEX-6P1 (amp-res) and RIP2 CARD (aa435–528) in pET-24b (kan-res) modified with a GB-1 (immunoglobulin binding domain of Streptococcal protein G) solubility tag were co-transformed into *E.coli* Rosetta 2 cells. Overexpression was performed as described above but in media supplemented with additional kanamycin. After cell lysis and centrifugation two overexpressed proteins were present in the soluble fraction: NOD2-CARDab fused to an N-terminal 3C-protease cleavable GST-tag and RIP2-CARD equipped with a C-terminal 6xHis-tag as well as an N-terminal uncleavable GB1-tag. The supernatant was loaded on gluthatione sepharose beads equilibrated with lysis buffer pH 8.0 and after washing the beads were boiled in SDS loading buffer and analyzed by SDS–polyacrylamide gel electrophoresis. Alternatively, the GST tag was cleaved on-column with Precission protease and the eluate analyzed by SDS-PAGE. The co-expression system was also used to study RIP2-CARD interactions with NOD1 CARD (aa17–138) and caspase-9 CARD (aa1–112), both expressed as N-terminal GST-fusion proteins. Control experiments were performed by co-expression of the GB-1 tag alone in pET24b with the NOD2-CARDab tandem construct as well as with the pGEX-6P1 vector. No interaction was detected between the GB-1 tag and NOD2-CARDab or between GB-1 and GST.

## Supporting Information

Figure S1 **Control gels for GST-pull down experiments.** (A) RIP2 CARD with a GB1 tag (RIP2-GB1) does not bind GST. Lane 1) Protein Marker, Sigma. Lane 2) Supernatant. RIP2-GB1 co-expressed with GST only. GST 26 kDa and RIP2-GB1 18.6 kDa are indicated by arrows. Lane 3) Supernatant bound to beads. Only GST is present. (B) The GB1 tag does bind GST-NOD2-CARDab. Lane 1) Protein Marker, GE Healthcare. Lane 2) Supernatant. GB1 8.6 kDa is indicated by arrow. Lane 3) Supernatant bound to beads. GST-NOD2-CARDab 48.5 kDa and GST 25.5 kDa are indicated by arrows. No GB1 is present.
(TIF)
