# Supplementary figures and images for: The Tandem CARDs of NOD2: Intramolecular Interactions and Recognition of RIP2
Source: PLoS One. 2012 Mar 28;7(3):e34375. doi: 10.1371/journal.pone.0034375 (PMC3314614; doi:10.1371/journal.pone.0034375)

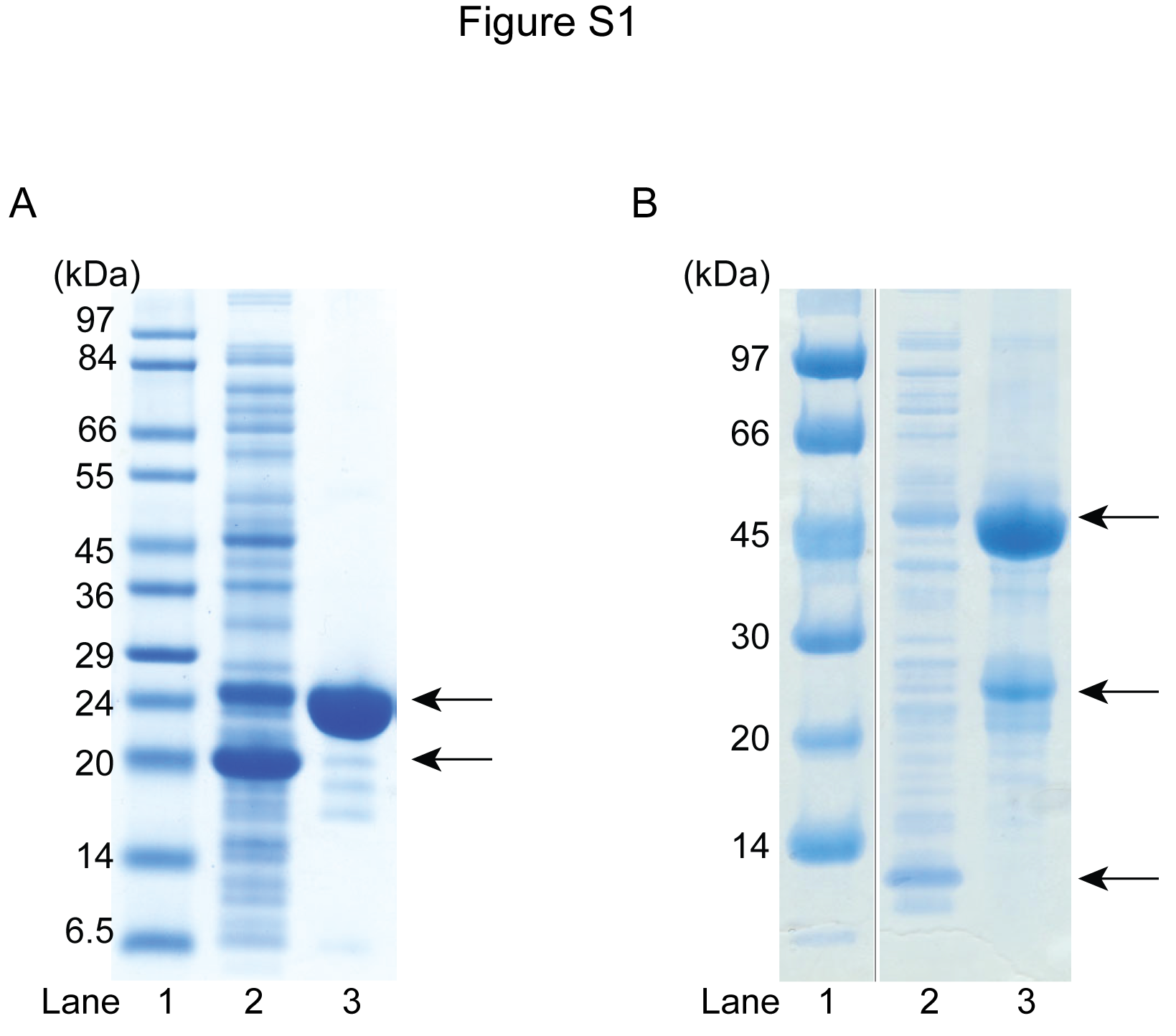

Supplement: Figure S1 — Control gels for GST-pull down experiments. (A) RIP2 CARD with a GB1 tag (RIP2-GB1) does not bind GST. Lane 1) Protein Marker, Sigma. Lane 2) Supernatant. RIP2-GB1 co-expressed with GST only. GST 26 kDa and RIP2-GB1 18.6 kDa are indicated by arrows. Lane 3) Supernatant bound to beads. Only GST is present. (B) The GB1 tag does bind GST-NOD2-CARDab. Lane 1) Protein Marker, GE Healthcare. Lane 2) Supernatant. GB1 8.6 kDa is indicated by arrow. Lane 3) Supernatant bound to beads. GST-NOD2-CARDab 48.5 kDa and GST 25.5 kDa are indicated by arrows. No GB1 is present. (TIF) [file pone.0034375.s001.tif]
